# The association of urine osmolality with decreased kidney function and/or albuminuria in the United States

**DOI:** 10.1186/s12882-021-02478-9

**Published:** 2021-09-10

**Authors:** Boonsong K. Kitiwan, Sarinnapha M. Vasunilashorn, Heather J. Baer, Kenneth Mukamal, Stephen P. Juraschek

**Affiliations:** 1 Department of Epidemiology, Harvard T.H. Chan School of Public Health, Boston, MA USA; 2 Department of Medicine, Division of Nephrology, Appalachian Regional Healthcare (ARH) Regional Medical Center, 200 Medical Center Drive, Hazard, KY 41701 USA; 3 Harvard Medical School, Boston, MA USA; 4 Department of Medicine, Division of General Medicine, Beth Israel Deaconess Medical Center, Boston, MA USA; 5 Department of Medicine, Division of General Internal Medicine and Primary Care, Brigham and Women’s Hospital, Boston, MA USA

**Keywords:** Urine osmolality, Hypovolemia, Decreased kidney function, Albuminuria

## Abstract

**Background:**

Decreased kidney function is commonly caused by hypovolemia. When hypovolemic, the kidney reabsorbs water resulting in concentrated urine. Osmolality is a measure of urine concentration which is more objective than self-reported fluid intake. It has a positive association with hypovolemia. However, it remains controversial whether osmolality is associated with decreased kidney function and/or albuminuria.

**Methods:**

We conducted a cross-sectional analysis of the 2009–2012 National Health and Nutrition Examination Survey, a standardized survey in the U.S. population. Participants aged 18–70 years old with random urine osmolality were included. Osmolality was categorized as quartiles. Decreased kidney function was defined by estimated glomerular filtration rate (eGFR) < 60 mL/min/1.73m^2^ and albuminuria was defined by albumin-to-creatinine ratio ≥ 30 mg/gm. We performed multivariable regression via four sequential models.

**Results:**

Our study sample included 7,373 participants. The mean age was 42.9 ± 0.4 years. Overall, 51.4% were male and 67.3% were white. The mean osmolality was 603.8 mOsm/kg and 629.1 mOsm/kg in those with and without decreased eGFR and/or albuminuria, respectively. The number of cases was 610 (6.7%). The prevalence from the lowest to highest quartiles of osmolality was 116 (6.2%), 213 (8.6%), 179 (7.5%), and 102 (4.3%), respectively (*p*-value for trend = 0.02). The relationship between osmolality and eGFR appeared nonlinear. After adjustment for demographic, social, cardiovascular, and dietary risk factors, there was no significant association of osmolality quartiles with decreased eGFR and/or albuminuria (odds ratio [OR] 0.77, 95% confidence interval [CI] 0.56, 1.07). In sensitivity analyses, osmolality ≥ 500 mOsm/kg was associated with lower eGFR (*adjusted ß* -1.13, 95% CI -1.98, -0.28). In pre-specified subgroup analyses, osmolality had a statistically significant negative correlation with eGFR among individuals with eGFR ≥ 60 mL/min/1.73m^2^, but a positive correlation among those with eGFR < 60 mL/min/1.73m^2^ (*adjusted ß* -0.19, 95% CI -0.36, -0.01 versus *adjusted ß* 0.50, 95% CI 0.05, 0.96; *p*-value for interaction = 0.016).

**Conclusions:**

Higher osmolality was significantly associated with lower eGFR among adults with eGFR ≥ 60 mL/min/1.73m^2^ Future research should examine the relationship between osmolality and change in kidney function over time among adults with normal eGFR.

**Supplementary Information:**

The online version contains supplementary material available at 10.1186/s12882-021-02478-9.

## Background 

It has been well known that hypovolemia, a condition when fluid intake and fluid loss are imbalanced, is one of the most common causes of an abrupt decline in kidney function, called *prerenal acute kidney injury* (AKI). Prolonged or severe intravascular volume depletion can result in lasting damage to renal tubules, particularly at the S3 segment of the proximal tubule and thick ascending limb of the loop of Henle in the outer medullary regions [1, 2], called *ischemic acute tubular necrosis* [3]. Recurrent episodes of hypovolemia-associated AKI cause kidney damage to accumulate over time, resulting in chronic kidney disease (CKD). *Mesoamerican nephropathy* is one example of CKD caused by hypovolemia among agricultural workers, primarily in Central America [4–8].

Urine osmolality, a measure of urine concentration, is the number of dissolved particles per unit of water. It is more objective than self-reported fluid intake. Moreover, it is more accurate than the use of urine specific gravity which can be affected by relatively large molecular masses such as glucose, contrast dye, or excessive protein. When hypovolemic, the posterior pituitary gland secretes antidiuretic hormone (ADH) or vasopressin, a hormone made by the hypothalamus, to direct the kidney to conserve water and concentrate urine through the osmotic gradient generated by countercurrent multiplication in the loop of Henle of juxtamedullary nephrons. Therefore, high urine osmolality has a physiologically positive association with hypovolemia.

Previous observational studies using urine osmolality have been inconsistent with some showing a relationship between high urine osmolality and decreased kidney function [9, 10], and others showing low urine osmolality being associated with kidney tubular damage [11–13]. However, these studies have been small and limited to populations with CKD [9–13].

Thus, in this study, we sought to examine the association between urine osmolality and decreased kidney function and/or albuminuria in a sample of the adult U.S. population. We hypothesized that high urine osmolality would be associated with decreased kidney function and/or albuminuria.

## Methods

### Study design and data source

We conducted a cross-sectional analysis of the 2009–2012 continuous National Health and Nutrition Examination Survey (NHANES), all of the cycles with measured urine osmolality. The NHANES are standardized, community-based surveys conducted by the Centers for Disease Control and Prevention (CDC) in the U.S. population aged 2 months and older. These surveys  collect demographic and clinical data through face-to-face home interviews, physical examination, and laboratory testing at mobile examination centers using well-validated methods. They are conducted annually with a complex sampling design administered within strata representative of age, sex, and race/ethnicity distributions of the U.S. population. The data are collected in 2-year cycles.

### Study population

**Fig. 1:**
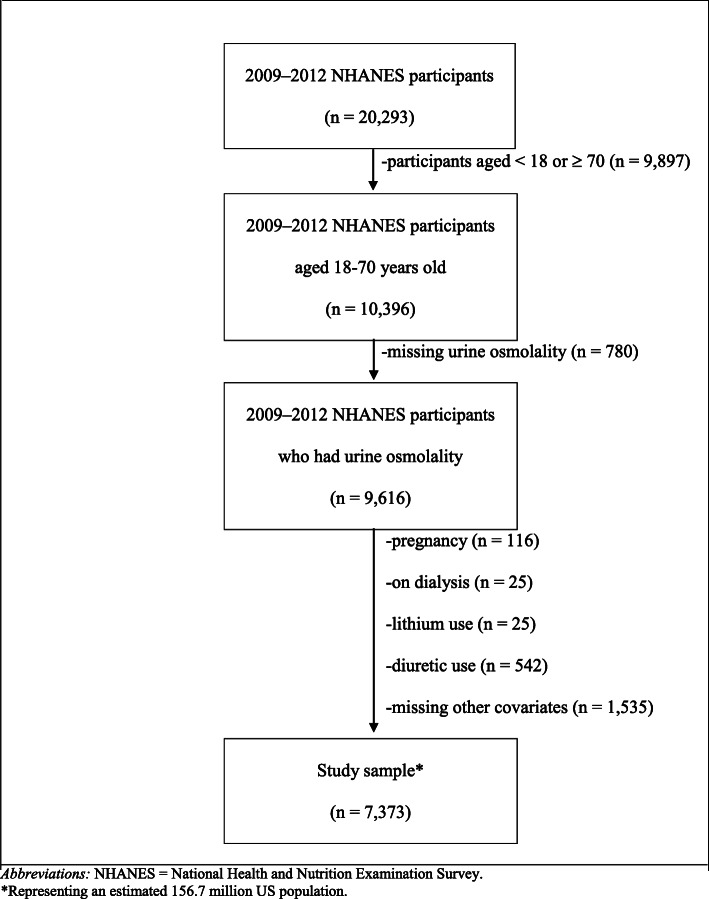
Study population

During the 2009–2012 cycles, 20,293 non-institutionalized participants were interviewed. The response rates for interviews were 75.87% [14, 15]. We restricted the analysis to participants aged ≥ 18 and < 70 years (*n* = 10,396) due to the limitation of creatinine-based equations for estimated glomerular filtration rate (eGFR) [16]. Among 9,616 participants who had urine osmolality measured, we excluded participants who were pregnant based on self-reported pregnancy at exam or a positive urine pregnancy test (*n* = 116) since pregnancy may affect eGFR levels. We also excluded those who received dialysis in the past 12 months (*n* = 25) and those who reported use of lithium (*n* = 25) or diuretics (including thiazides, loop diuretics, and potassium-sparing agents) during a 30-day period prior to the survey date (*n* = 542), because these may interfere with the interpretation of urine osmolality. In addition, we excluded 1,535 participants who were missing other covariates of interest including education, smoking, body mass index (BMI), diabetes, hypertension, coronary artery disease, and sodium intake. Our final study sample included 7,373 participants, representing an estimated 156.7 million people (Fig. 1).

### Exposures

The primary exposure was random urine osmolality, collected at the mobile exam center and measured by freezing point determination (OSMETTE II^TM^ Model 5005, Automatic Osmometer, Precision Systems, Inc., Natick, MA, USA). Detailed instructions on specimen collection and processing can be found in the NHANES Laboratory/Medical Technologists Procedures Manual [17].

Urine osmolality was categorized as quartiles. In sensitivity analyses, we also treated urine osmolality as a continuous variable in a 100-milliosmole per kilogram (mOsm/kg) increment and binary variables using cut-off values of ≥ 500 and ≥ 800 mOsm/kg based on population distributions used to define inadequate hydration in previous studies [18–20].

### Outcomes

The primary outcome was decreased eGFR < 60 mL/min/1.73m^2^ and/or albuminuria, defined by a urine albumin-to-creatinine ratio (ACR) ≥ 30 miligrams per gram (mg/gm). Blood and urine samples were collected on the same day. An eGFR was measured from isotope dilution mass spectrometry (IDMS) traceable serum creatinine and it was calculated from the CKD Epidemiology Collaboration (CKD-EPI) equation [21]. The secondary outcomes included eGFR < 60 mL/min/1.73m^2^, eGFR, albuminuria, and log-transformed ACR. We used the log-transformed ACR to normalize the distribution of the albuminuria residuals to address a right-tailed skew.

### Covariates

Based on a priori knowledge, we adjusted for *demographic factors* including age, sex, and race/ethnicity (non-Hispanic white, non-Hispanic black, Mexican American, Hispanic, and others); *social factors* including education (less than high school diploma, high school diploma, and some college or higher), family income-to-poverty ratio (below the federal poverty threshold if ratio < 1, above if otherwise), and smoking (current smokers, former smokers if smoke > 100 in lifetime, but not current, and never smokers if not current and less than 100 in lifetime). *Cardiovascular risk factors* comprised BMI (obese if BMI ≥ 30 kg/m^2^, overweight if BMI ≥ 25 to < 30 kg/m^2^, and underweight to normal if < 25 kg/m^2^) [22, 23]; diabetes including borderline diabetes based on self-report, use of diabetes medications, or hemoglobin A1C ≥ 6.5% [24]; hypertension based on self-report, antihypertensive medication use, mean systolic blood pressure ≥ 130 mmHg, or mean diastolic blood pressure ≥ 80 mmHg [25]; and self-reported coronary heart disease.

Furthermore, we adjusted for one *dietary factor*, self-reported sodium consumption, based on a mean of two 24-hour dietary recalls of sodium intake (mg/day) in a 500-mg (equivalent to quarter a teaspoon salt) increment. We focused on sodium as this is the primary determinant of urine osmolality from diet.

### Statistical analysis

All analyses were weighted using the mobile examination center weights following recommendations provided by the CDC [26]. Taylor linearization and the NHANES stratification scheme were used to generate robust linearized standard errors according to the National Center for Health Statistics (NCHS) recommendations with further details described in the NHANES Analytic Guidelines [27].

We visualized the relationship between urine osmolality and eGFR and the relationship with log ACR by using locally weighted scatterplot smoothing (LOWESS) curves. We characterized our population via weighted means and proportions.

In primary analyses, we examined the relationship of osmolality quartiles with eGFR < 60 mL/min/1.73m^2^ and/or albuminuria, using unadjusted and multivariable logistic regression models via four sequential models. *Model 1* was adjusted for demographic factors as described above. *Model 2* included Model 1 plus social factors. *Model 3* included Model 2 plus cardiovascular risk factors. *Model 4* included Model 3 plus our dietary factor. In sensitivity analyses, models were also repeated treating urine osmolality as a continuous variable and a dichotomous variable, respectively.

In secondary analyses, we used similar logistic regression models to examine the relationship between urine osmolality and eGFR < 60 mL/min/1.73m^2^ and/or albuminuria. We also used linear regression models adjusted in the same fashion as above to examine the relationship between urine osmolality and eGFR and log-transformed ACR. In exploratory analyses, we further examined the association of urine osmolality in a 100-mOsm/kg increment with eGFR and log-transformed ACR in subpopulations with (a) eGFR < 60 mL/min/1.73m^2^ versus eGFR ≥ 60 mL/min/1.73m^2^ and (b) albuminuria ≥ 30 mg/gm versus no albuminuria < 30 mg/gm by using unadjusted and multivariable regression models with interaction terms. Moreover, we examined the association between osmolality quartiles with eGFR < 60 mL/min/1.73m^2^ and/or albuminuria in subgroups of age (< 40, 40–60, ≥ 60 years), sex, race, diabetes, and hypertension using the fully adjusted logistic regression models described above. Differences in associations were compared across strata using interaction terms. Complete case analysis was performed to handle missing data.

All analyses were performed using Stata version 15.1 (College Station, TX, USA; StataCorp LLC). *P*-values < 0.05 were considered statistically significant. We confirm that all methods were carried out in accordance with relevant guidelines and regulations.

## Results

**Fig. 2:**
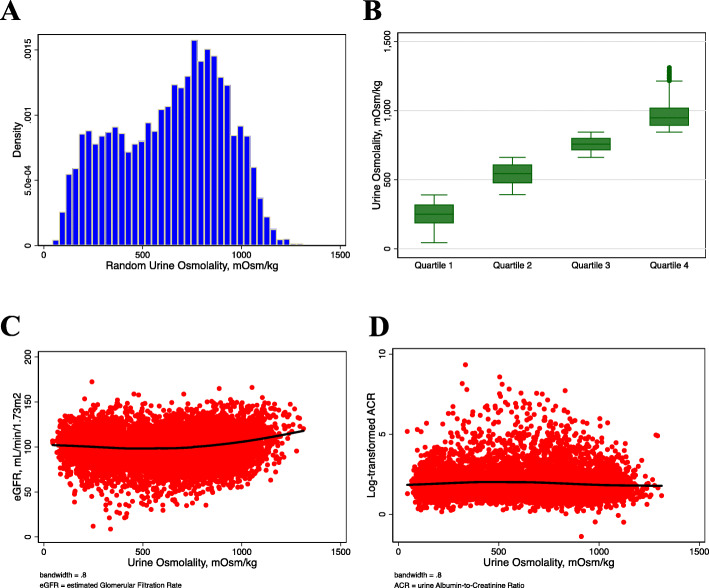
**A** Distribution of urine osmolality, **B** Distribution of urine osmolality quartiles, **C** A LOWESS curve showing a relationship between urine osmolality and eGFR, and **D**. A LOWESS curve showing relationship between urine osmolality and log-transformed ACR

The mean age of the sample was 42.9 ± 0.4 years. Overall, 51.4% were male and 67.3% were non-Hispanic white. The mean osmolality was 627.4 ± 5.9 mOsm/kg (Fig. 2a). It was 603.8 mOsm/kg and 629.1 mOsm/kg in those with and without decreased eGFR and/or albuminuria, respectively (Supplement Figure SF1). The mean urine osmolality in the lowest to highest quartiles was 250.0, 540.5, 756.9, and 963.0 mOsm/kg, respectively (Fig. 2b). Participants with higher urine osmolality were more likely to be younger, male, non-white, obese, smokers, and have lower education and income. The mean eGFR was 98.8 mL/min/1.73m^2^ and 489 (5%) participants had albuminuria ≥ 30 mg/gm (Table 1).

**Table 1:** Baseline characteristics based on urine osmolality quartiles in 2009–2012 NHANES participants

Characteristic		Urine Osmolality (mOsm/kg)			
	Total Sample (*N* = 7373)	Quartile 1 (*N* = 1706)	Quartile 2 (*N* = 1811)	Quartile 3 (*N* = 1879)	Quartile 4 (*N* = 1977)
Urine osmolality (mOsm/kg)	627.4 (5.9)	250.0 (2.2)	540.5 (2.5)	756.9 (1.4)	963.0 (2.6)
Serum glucose (mg/dL)	96.4 (0.4)	92.2 (0.5)	97.3 (0.7)	99.4 (1.0)	96.7 (0.7)
Age (years)	42.9 (0.4)	43.6 (0.6)	46.2 (0.6)	43.9 (0.4)	38.1 (0.5)
Male (%)	51.4	40.4	50.8	53.4	61.1
Race/ethnicity (%)					
Non-Hispanic white	67.3	73.8	70.9	66.0	58.6
Non-Hispanic black	10.5	6.3	10.2	11.1	14.6
Mexican American	9.0	6.7	6.8	9.7	12.7
Hispanic	6.0	5.0	5.0	5.7	8.0
Other races	7.2	8.2	7.2	7.5	6.1
Education (%)					
< High school diploma	15.6	14.3	15.3	16.9	16.1
High school diploma	20.9	18.4	20.8	20.4	24.0
≥ Some college	63.5	67.3	64.0	62.8	59.9
Income-to-poverty (%)^a^					
< 1	15.2	14.4	12.6	15.8	18.0
Smoking (%)^b^					
Never	56.2	55.5	54.6	54.2	60.3
Former	21.9	20.2	23.6	25.4	18.5
Current	21.9	24.3	21.8	20.4	21.2
Sodium intake (mg/d)	3677.9 (25.7)	3475.6 (53.4)	3631.3 (60.1)	3752.6 (38.5)	3852.4 (45.0)
BMI (kg/m^2^) (%)^c^					
< 25	32.0	44.9	32.0	26.2	24.6
25–30	33.7	32.8	34.6	34.5	32.7
≥ 30	34.4	22.3	33.3	39.3	42.7
Diabetes (%)^d^	9.6	6.8	11.6	12.4	7.7
Hypertension (%)^e^	35.5	33.3	39.7	39.9	29.1
Coronary artery disease (%)	1.6	1.6	2.1	1.3	1.4
Decreased eGFR ± albuminuria (%)^f^	6.7	6.2	8.6	7.5	4.3
Decreased eGFR (%)	2.2	1.9	3.8	2.0	0.8
eGFR (mL/min/1.73m^2^)	98.8 (0.5)	99.0 (0.6)	95. 0 (0.7)	98.2 (0.7)	102.8 (0.7)
Albuminuria ≥ 30 mg/gm (%)^g^	5.0	4.7	5.7	6.0	3.5
Log-transformed ACR	1.9 (0.0)	1.9 (0.0)	1.9 (0.0)	1.9 (0.0)	1.8 (0.0)

*Abbreviations: NHANES* National Health and Nutrition Examination Survey, *SE* Standard Error, *BMI* Body Mass Index, *eGFR* estimated Glomerular Filtration Rate, *ACR* urine Albumin-to-Creatinine Ratio

Weighted data are expressed as the mean (SE) or percentage of participants. No random urine electrolytes in this publicly available dataset. No differences in serum sodium, potassium, and calcium in each osmolality quartile.

^a^ Income-to-poverty ratio < 1 if family income below the federal poverty threshold

^b^For self-reported smoking status, *current smokers* if currently smoking, *former smokers* if smoke > 100 in lifetime, but not current, and *never smokers* if not current and less than 100 in lifetime

^c^Obese if BMI ≥ 30 kg/m^2^, overweight if BMI ≥ 25 to < 30 kg/m^2^, and underweight to normal if < 25 kg/m^2^

^d^Diabetes was defined as 1) self-report including borderline diabetes by using the question “Have you ever been told by a doctor or health professional that you have diabetes?” 2) use of diabetes medications, or 3) HbA1C ≥ 6.5%

^e^Hypertension was defined as self-report, use of antihypertensive agents, mean systolic BP ≥ 130 mmHg, or mean diastolic BP ≥ 80 mmHg

^f^Decreased eGFR was defined by eGFR < 60 mL/min/1.73m^2^. Albuminuria was defined by ACR ≥ 30 mg/gm. The total unweighted number of cases was 610. The prevalence from the lowest to highest quartiles of osmolality was 116 (6.2%), 213 (8.6%), 179 (7.5%), and 102 (4.3%), respectively (*p*-value for trend = 0.02)

^g^Albuminuria was based on spot urine albumin-to-creatinine ratio

**Fig. 3:**
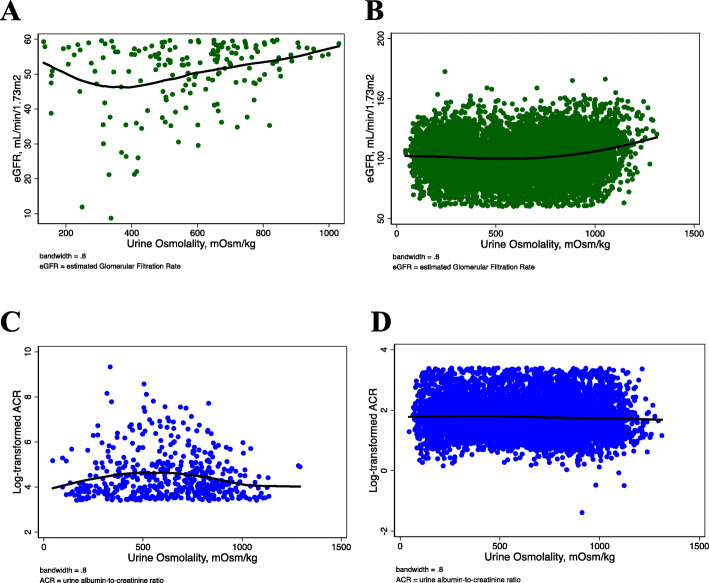
**A** A LOWESS curve showing a relationship between urine osmolality and eGFR in the subgroup with eGFR < 60 mL/min/1.73m^2^, **B** A LOWESS curve showing a relationship between urine osmolality and eGFR in the subgroup with eGFR ≥ 60 mL/min/1.73m^2^, **C** A LOWESS curve showing a relationship between urine osmolality and log-transformed ACR in the subgroup with albuminuria, **D** A LOWESS curve showing a relationship between urine osmolality and log-transformed ACR in the subgroup with no albuminuria

The total number of cases was 610 (6.7%), representing an estimated 10.4 million people. The prevalence from the lowest to highest quartiles of osmolality was 116 (6.2%), 213 (8.6%), 179 (7.5%), and 102 (4.3%), respectively (*p*-value for trend = 0.02). The relationship between urine osmolality and eGFR appeared curvilinear (Fig. 2c), while the relationship with log ACR was nearly flat (Fig. 2d). The curvilinear curve with eGFR was more convex, like a J shape in the subgroup with eGFR < 60 mL/min/1.73m^2^ (Fig. 3a-b), whereas the flat relationship with log ACR was slightly concave in the subgroup with albuminuria (Fig. 3c-d).

In the primary analyses, participants in the highest osmolality quartile had a 31% lower prevalence of decreased eGFR and/or albuminuria (odds ratio [OR] 0.69, 95% confidence interval [CI] 0.51, 0.93) when compared with the lowest osmolality quartile. However, after full adjustment for all of demographic, social, cardiovascular, and dietary risk factors, this association was no longer significant (OR 0.77, 95% CI 0.56, 1.07) (Table 2). In the sensitivity analyses treating urine osmolality as a continuous variable and binary variables, only urine osmolality ≥ 500 mOsm/kg was associated with lower kidney function (*adjusted ß* -1.13, 95% CI -1.98, -0.28) (Table 3).

**Table 2:** Unadjusted and adjusted weighted association with decreased eGFR and/or albuminuria among 7,373 NHANES participants

Variable	Urine Osmolality (mOsm/kg)			
	Quartile 1 (*N* = 1706)	Quartile 2 (*N* = 1811)	Quartile 3 (*N* = 1879)	Quartile 4 (*N* = 1977)
**Primary Outcome**				
**DecreasedeGFR** **±** **albuminuria** (unweighted cases, %)^a^	116 (6.2%)	213 (8.6%)	179 (7.5%)	102 (4.3%)
*Logistic Regression Analysis — Odds Ratio (95% Confidence Interval)*				
Unadjusted Analysis	Ref	1.42 (1.03, 1.96)	1.24 (0.92, 1.66)	0.69 (0.51, 0.93)
Multivariable Analysis				
Model 1^b^	Ref	1.23 (0.88, 1.73)	1.16 (0.88, 1.52)	0.82 (0.60, 1.11)
Model 2^c^	Ref	1.26 (0.90, 1.74)	1.17 (0.89, 1.53)	0.84 (0.63, 1.12)
Model 3^d^	Ref	1.17 (0.84, 1.64)	1.03 (0.78, 1.37)	0.77 (0.56, 1.06)
Model 4^e^	Ref	1.17 (0.84, 1.64)	1.04 (0.78, 1.37)	0.77 (0.56, 1.07)
**Secondary Outcomes**				
**DecreasedeGFR** (unweighted cases, %)	36 (1.9)	76 (3.8)	46 (2.0)	14 (0.8)
*Logistic Regression Analysis — Odds Ratio (95% Confidence Interval)*				
Unadjusted Analysis	Ref	2.09 (1.15, 3.80)	1.08 (0.52, 2.25)	0.43 (0.16, 1.18)
Multivariable Analysis				
Model 1^b^	Ref	1.67 (0.89, 3.14)	1.07 (0.52, 2.19)	0.80 (0.28, 2.29)
Model 2^c^	Ref	1.67 (0.93, 3.00)	1.01 (0.50, 2.03)	0.78 (0.27, 2.24)
Model 3^d^	Ref	1.53 (0.85, 2.76)	0.85 (0.42, 1.72)	0.65 (0.23, 1.81)
Model 4^e^	Ref	1.55 (0.86, 2.80)	0.86 (0.42, 1.76)	0.66 (0.23, 1.86)
**eGFR** (mL/min/1.73m^2^) — mean (SE)	99.0 (0.6)	95.0 (0.7)	98.2 (0.7)	102.8 (0.7)
*Linear Regression Analysis — ß (95% Confidence Interval)*				
Unadjusted Analysis	Ref	−4.00 (−5.55, −2.46)	−0.86 (−2.60, 0.87)	3.80 (2.27, 5.32)
Multivariable Analysis				
Model 1^b^	Ref	−1.90 (−2.85, −0.95)	−0.90 (−2.17, 0.36)	−1.25 (−2.48, −0.02)
Model 2^c^	Ref	−1.87 (−2.79, −0.94)	−0.87 (−2.08, 0.34)	−1.17 (−2.31, −0.02)
Model 3^d^	Ref	−1.87 (−2.79, −0.95)	−0.88 (−2.06, 0.30)	−1.12 (−2.28, 0.05)
Model 4^e^	Ref	−1.88 (−2.80, −0.96)	−0.91 (−2.10, 0.28)	−1.14 (−2.28, 0.01)
**Albuminuria** (unweighted cases, %)^f^	94 (4.7)	166 (5.7)	140 (6.0)	89 (3.5)
*Logistic Regression Analysis — Odds Ratio (95% Confidence Interval)*				
Unadjusted Analysis	Ref	1.24 (0.84, 1.82)	1.28 (0.94, 1.75)	0.74 (0.51, 1.09)
Multivariable Analysis				
Model 1^b^	Ref	1.10 (0.74, 1.64)	1.17 (0.86, 1.59)	0.75 (0.50, 1.13)
Model 2^c^	Ref	1.12 (0.76, 1.66)	1.18 (0.85, 1.64)	0.78 (0.52, 1.17)
Model 3^d^	Ref	1.05 (0.71, 1.56)	1.05 (0.75, 1.47)	0.73 (0.48, 1.13)
Model 4^e^	Ref	1.07 (0.73, 1.57)	1.09 (0.78, 1.51)	0.76 (0.51, 1.14)
**Log-transformed ACR** — mean (SE)	1.9 (0.0)	1.9 (0.0)	1.9 (0.0)	1.8 (0.0)
*Linear Regression Analysis — ß (95% Confidence Interval)*				
Unadjusted Analysis	Ref	0.02 (−0.06, 0.10)	−0.03 (−0.11, 0.05)	−0.13 (−0.21, −0.05)
Multivariable Analysis				
Model 1^b^	Ref	0.02 (−0.06, 0.10)	−0.01 (−0.09, 0.07)	−0.06 (−0.14, 0.02)
Model 2^c^	Ref	0.02 (−0.05, 0.10)	−0.01 (−0.08, 0.07)	−0.05 (−0.13, 0.02)
Model 3^d^	Ref	0.01 (−0.06, 0.09)	−0.02 (−0.10, 0.06)	−0.05 (−0.13, 0.03)
Model 4^e^	Ref	0.01 (−0.06, 0.09)	−0.02 (−0.10, 0.06)	−0.05 (−0.13, 0.03)

*Abbreviations: NHANES* National Health and Nutrition Examination Survey, *SE* Standard Error, *eGFR* estimated Glomerular Filtration Rate, *ACR* urine Albumin-to-Creatinine Ratio.

^a^Decreased eGFR was defined by eGFR < 60 mL/min/1.73m^2^. Albuminuria was defined by ACR ≥ 30 mg/gm. The total unweighted number of cases was 610. The prevalence from the lowest to highest quartiles of osmolality was 116 (6.2%), 213 (8.6%), 179 (7.5%), and 102 (4.3%), respectively (*p*-value for trend = 0.02).

^b^Model 1 was adjusted for demographic factors including age, sex, and race/ethnicity (non-Hispanic white, non-Hispanic black, Mexican American, Hispanic, and other races).

^c^Model 2 was Model 1 plus social factors including education (< high school, high school, and ≥ college), family income-to-poverty (< 1 vs ≥ 1), and smoking (never, former, and current).

^d^Model 3 was Model 2 plus cardiovascular risk factors including BMI (< 25, 25–30, and ≥ 30 kg/m^2^), diabetes, hypertension, and coronary artery disease.

^e^Model 4 was Model 3 plus a dietary sodium intake (500-mg increment or quarter a teaspoon salt)

^f^Albuminuria was based on spot urine albumin-to-creatinine ratio ≥ 30mg/gm

**Table 3:** Multivariable linear and logistic regression models on decreased eGFR and/or albuminuria^a^

Exposure	Primary Outcome	Secondary Outcomes			
	**Decreased eGFR ± albuminuria**^**b**^	**Decreased eGFR**	**eGFR**	**Albuminuria**	**Log ACR**
	(*N* = 613, 6.7%)	(*N* = 172, 2.2%)	(mL/kg/1.73m^2^)	(*N* = 492, 5.0%)	(log [mg/gm])
	OR (95% CI)	OR (95% CI)	*ß* (95% CI)	OR (95% CI)	*ß* (95% CI)
**Urine Osmolality** (mOsm/kg)^c^					
Continuous	0.98 (0.95, 1.01)	0.93 (0.86, 1.01)	−0.13 (−0.29, 0.02)	0.98 (0.94, 1.02)	−0.01 (−0.02, 0.00)
< 500 [20]			Reference		
≥ 500	1.02 (0.82, 1.26)	0.93 (0.63, 1.39)	−1.13 (−1.98, −0.28)	0.97 (0.73, 1.30)	−0.03 (−0.10, 0.03)
< 800 [18, 19]			Reference		
≥ 800	0.92 (0.71, 1.18)	0.64 (0.31, 1.35)	0.04 (−0.85, 0.93)	0.97 (0.73, 1.29)	−0.02 (−0.07, 0.03)

*Abbreviations: eGFR* estimated Glomerular Filtration Rate, *ACR* urine Albumin-to-Creatinine Ratio, *OR* Odds Ratio, *CI* Confidence Interval

^a^*Fully-adjusted* for age, sex, race/ethnicity, education, family income-to-poverty ratio, smoking, BMI, diabetes, hypertension, coronary heart disease, and dietary sodium intake

^b^Decreased eGFR was defined by eGFR < 60 mL/min/1.73m^2^. Albuminuria was defined by ACR ≥ 30 mg/gm

^c^A 100-mOsm/kg increment

Similarly, in the secondary analyses, urine osmolality was not associated with any of the secondary outcomes including decreased eGFR and albuminuria after the full adjustment as previously mentioned (OR 0.66, 95% CI 0.23, 1.86; OR 0.76, 95% CI 0.51, 1.14, respectively). In exploratory pre-specified subgroup analyses, urine osmolality had a statistically significant negative association with eGFR in the subgroup with eGFR ≥ 60 mL/min/1.73m^2^ (*N* = 7,201, 97.7%; mean age 42.6 ± 0.4 years; 51.6% male; mean urine osmolality 628.9 ± 6.0 mOsm/kg; mean eGFR 99.8 ± 0.4 mL/min/1.73m^2^), but a positive association in the subgroup with eGFR < 60 mL/min/1.73m^2^ (*N* = 172, 2.3%; mean age 59.5 ± 0.6 years; 45.6% male; mean urine osmolality 559.2 ± 21.8 mOsm/kg; mean eGFR 52.0 ± 0.6 mL/min/1.73m^2^) after full adjustment (*ß* -0.19, 95% CI -0.36, -0.01 vs *ß* 0.50, 95% CI 0.05, 0.96; *p*-value for interaction = 0.016) (Table 4).

**Table 4:** Subgroup analyses examining the association of urine osmolality with eGFR and log ACR

Exposure	Kidney function/injury					
	**eGFR** (mL/min/1.73m^2^)			**Log-transformed ACR**		
	Unadjusted	Multivariable^a^	*p*-value*	Unadjusted	Multivariable^a^	*p*-value*
	*ß* (95% CI)	*ß* (95% CI)		*ß* (95% CI)	*ß* (95% CI)	
**Urine Osmolality** (mOsm/kg)^b^						
**eGFR** (mL/min/1.73m^2^)						
**< 60**	0.64 (-0.05, 1.33)	0.50 (0.05, 0.96)	0.016	-0.06 (-0.20, 0.07)	-0.06 ( -0.21, 0.09)	0.532
(*N* = 172, 2.3%; mean 52.0 ± 0.6 mL/min/1.73m^2^)						
**≥ 60**	0.46 (0.23, 0.68)	-0.19 ( -0.36, -0.01)		-0.01 (-0.02, -0.00)	-0.00 (-0.01, 0.01)	
(*N* = 7,201, 97.7%; mean 99.8 ± 0.4 mL/min/1.73m^2^)						
**Albuminuria** (mg/gm)^c^						
**Yes**	2.03 (1.07, 2.99)	0.43 ( -0.33, 1.20)	0.060	-0.02 (-0.06, 0.02)	-0.05 (-0.09, -0.00)	0.959
(*N* = 489, 6.6%; mean 169.3 ± 20.7 mg/gm)						
**No**	0.48 (0.24, 0.71)	-0.17 (-0.33, -0.01)		-0.01 (-0.02, -0.01)	-0.00 (-0.01, 0.01)	
(*N* = 6,884, 93.4%; mean 6.8 ± 0.1 mg/gm)						

*Abbreviations: eGFR* estimated Glomerular Filtration Rate, *ACR* urine Albumin-to-Creatinine Ratio, *CI* Confidence Interval

^a^*Fully-adjusted* for age, sex, race/ethnicity, education, family income-to-poverty ratio, smoking, BMI, diabetes, hypertension, coronary heart disease, and dietary sodium intake

^b^A 100-mOsm/kg increment

^c^Albuminuria was based on spot urine albumin-to-creatinine ratio ≥ 30 mg/gm

*p-values for interaction

Findings were consistent across subgroups without evidence of effect modification (Supplement Table ST1).

## Discussion

In this sample of the U.S. population, the association between osmolality quartiles and decreased eGFR and/or albuminuria was not significant. However, higher urine osmolality was significantly associated with lower eGFR in adults with eGFR ≥ 60 mL/min/1.73m^2^. On the other hand, urine osmolality was positively associated with greater kidney function in adults with eGFR ≤ 60 mL/min/1.73m^2^.

This is the first study using urine osmolality from the NHANES data to examine the association with decreased eGFR and/or albuminuria in a general population. Previous studies only examined this association in CKD population. The post hoc analysis of the Modification of Diet in Renal Disease (MDRD) study (*N* = 581; baseline eGFR 25–55 mL/min/1.73 m^2^) by Hebert *et al*. in 2003 demonstrated low urine osmolality is an independent risk factor for reduced kidney function regardless of polycystic kidney disease (PKD) [11]. Recently, two prospective studies using urine osmolality tertiles, conducted in 2019 by the French NephroTest Study Group [12] (*N* = 2,084; mean age 58.7 ± 15.2 years; 67.7% male; mean urine osmolality 502.7 ± 151.7 mOsm/kg; median baseline GFR 40.2 mL/min/1.73m^2^ with interquartile range of 29.1–54.5) and Lee *et al.* from the Korean Cohort Study on the Outcome of Chronic Kidney Disease Patients (KNOW-CKD) [13] (*N* = 1,999; mean age 53.8 ± 12.1 years; 61% males; mean baseline eGFR 50.3 ± 30.0 mL/min/1.73m^2^) demonstrated that low urine osmolality was associated with a higher risk of kidney impairment and there was a significant interaction between urine osmolality and eGFR. These are consistent with the findings in our subgroup with eGFR < 60 mL/min/1.73m^2^ (*N* = 172, 2.3%; mean age 59.5 ± 0.6 years; 45.6% male; mean urine osmolality 559.2 ± 21.8 mOsm/kg; mean eGFR 52.0 ± 0.6 mL/min/1.73m^2^).

Urine osmolality is dynamic as the body corrects temporary water imbalance and can help evaluate renal concentrating ability, and fluid balance between fluid intake and fluid loss. As a determination of the amount of osmotically active solutes in the urine, (a) *any factors increasing urine output* including increased fluid intake, water diuresis from diabetes insipidus, osmotic diuresis from hyperglycemia, urea diuresis from improving AKI, and sodium diuresis from high salt intake, or (b) *any factors decreasing urine output* such as hypovolemia, decreased renal blood flow, and damage to renal tubular cells can impact urine osmolality. Moreover, calculating solute excretion is useful to determine the type of diuresis.

Our findings support the hypothesis that high urine osmolality is associated with decreased eGFR in our healthier sample with eGFR ≥ 60 mL/min/1.73m^2^ and it can be explained by (a) *hypovolemia* which refers to a reduction of effective circulating volume with salt and water loss via gastrointestinal system, diuretics, bleeding, or third space sequestration, or (b) *dehydration* which refers to a reduction of total body water with water loss (as with diabetes insipidus) or inadequate water intake, resulting in a rise in the plasma sodium, called *hypernatremia*. To support this hypothesis, urine output as well as urine sodium and creatinine would be required. If urine osmolality is high and urine output decreases, hypovolemia is likely. If urine osmolality is high but urine output increases, then it could be due to osmotic diuresis. However, these mechanisms are beyond the data available in our dataset.

In addition, we observed the positive relationship in our sample with eGFR < 60 mL/min/1.73m^2^. This could be because the predictive ability of urine osmolality depends on renal concentrating ability. Biologically plausible mechanisms of a reduction in maximal urine concentrating ability in CKD have been proposed. Low urine osmolality can directly cause CKD by increasing the intratubular urine volume and pressure leading to fibrosis [11, 13]. Conversely, damage to tubular cells in CKD, particularly in PKD might result in a decline urine osmolality [11] and an increase in vasopressin, leading to progression of CKD [13, 28]. Moreover, the concentrating ability can be compromised by older age, which is commonly observed in CKD populations.

Our study has some limitations. As a cross-sectional study, causality cannot be determined, and residual confounding is always a concern. After the exclusion criteria, our sample was technically no longer nationally representative. Although random urine osmolality is clinically feasible, it is less accurate than 24-hour urine collection due to the diurnal variation of urine osmolality. Moreover, urine osmolality reflects the amount of fluid intake and the number of osmotically active solutes; therefore, it can be impacted by several factors such as fasting or non-fasting measurements, and dietary protein and salt intake. Urine osmolality should be used in conjunction with the urine flow rate to assess solute excretion. However, it had substantial missing data. Therefore, this parameter was not presented. Missing data may elicit biased results; however, missingness excluded in our study was less than 10%. Finally, the study probably has low power to detect interactions, thus the interaction analyses are exploratory.

Our study has several strengths. This is the first study examining the association between urine osmolality and kidney function using NHANES data. This large nationwide database provides comprehensive data from in-person interviews, physical exams, and lab testing by trained personnel allowing us to robustly adjust for potential confounders, particularly when randomized controlled trials, the gold standard for causation, are difficult to perform. Random urine osmolality is more precise than self-reported fluid intake in predicting hydration status since it entails fluid loss to reflect fluid balance in the body and is less subject to recall bias. CKD is associated with low or normal osmolality rather than high osmolality due to renal concentrating defects. Therefore, reverse causation is unlikely. Furthermore, our large sample is more broadly generalizable than the prior reports.

## Conclusions

We found that higher urine osmolality was significantly associated with lower eGFR in adults with eGFR ≥ 60 mL/min/1.73m^2^, but it was associated with greater kidney function in adults with eGFR < 60 mL/min/1.73m^2^.

Urine osmolality is not a diagnostic test and must be evaluated with clinical presentation and other laboratory findings. It may be useful to suggest a fluid imbalance when renal concentrating ability is intact, but does not identify the cause; therefore, kidney function and renal tubular damage, such as PKD, should be taken into account in conjunction with the interpretation of urine osmolality. In adults with CKD, urine osmolality may not be reliable. Future longitudinal studies using a baseline fasting 24-hour urine osmolality and subsequent kidney outcomes in a large general population without CKD are warranted.

## Supplementary Information

**Additional file 1: Table ST1.** Subgroup analyses examining the association between urine osmolality quartiles with decreased eGFR and/or albuminuria. **Figure SF1.** **A** Distribution of urine osmolality by the presence of decreased eGFR and/or albuminuria, **B** Distribution of urine osmolality by eGFR levels.

## Data Availability

The datasets generated and/or analyzed during the current study are available in the 2009–2012 continuous National Health and Nutrition Examination Survey. Please see https://wwwn.cdc.gov/nchs/nhanes/

## Abbreviations

CKD: Chronic Kidney Disease
AKI: Acute Kidney Injury
eGFR: estimated Glomerular Filtration Rate
ACR: Albumin-to-Creatinine Ratio
BMI: Body Mass Index
PKD: Polycystic Kidney Disease
NHANES: National Health and Nutrition Examination Survey
CKD-EPI: Chronic Kidney Disease Epidemiology Collaboration
