# Supplementary material for: The association of urine osmolality with decreased kidney function and/or albuminuria in the United States
Source: BMC Nephrol. 2021 Sep 10;22:306. doi: 10.1186/s12882-021-02478-9 (PMC8434733; doi:10.1186/s12882-021-02478-9)

**The association of urine osmolality with decreased kidney function and/or albuminuria in the United States**

Boonsong K. Kitiwan, MD, MPH;^1,2^ **Sarinnapha M. Vasunilashorn, PhD;^1,3,4^** Heather J. Baer, ScD;^1,3,5^ Kenneth Mukamal, MD, MPH;^3,4^ and Stephen P. Juraschek, MD, PhD^3,4^

1. Department of Epidemiology, Harvard T.H. Chan School of Public Health, Boston, MA, USA
2. Department of Medicine, Division of Nephrology, Appalachian Regional Healthcare (ARH) Regional Medical Center, Hazard, KY, USA
3. Harvard Medical School, Boston, MA, USA
4. Department of Medicine, Division of General Medicine, Beth Israel Deaconess Medical Center, Boston, MA, USA
5. Department of Medicine, Division of General Internal Medicine and Primary Care, Brigham and Women’s Hospital, Boston, MA, USA

Author last names for PubMed indexing: Kitiwan, Vasunilashorn, Baer, Mukamal, Juraschek

Corresponding Author:

Boonsong K. Kitiwan, MD, MPH, FACP, FASN

ARH Nephrology Clinic

200 Medical Center Drive

Hazard, KY, 41701

Phone: (606) 487-7673

Fax: (606) 439-6879

E-mail: [bkitiwan@alumni.harvard.edu](mailto:bkitiwan@alumni.harvard.edu)

**Supplement Material**

**Supplement Table ST1.** Subgroup analyses examining the association between urine osmolality quartiles with decreased eGFR and/or albuminuria

**Supplement Figure SF1.** **A** Distribution of urine osmolality by the presence of decreased eGFR and/or albuminuria, **B** Distribution of urine osmolality by eGFR levels

| **Table ST1** Subgroup analyses examining the association between urine osmolality quartiles with decreased eGFR and/or albuminuria |
| --- |
| **Subgroups** **Urine Osmolality** (mOsm/kg)  **Quartile 1 Quartile 2 Quartile 3 Quartile 4 *p*-value***  (N = 1,706) (N = 1,811) (N = 1,879) (N = 1,977) |
| **Decreased eGFR ± albuminuria**†  *Adjusted* *Logistic Regression Analysis — Odds Ratio (95% Confidence Interval)*°  **Age groups** (years)‡  Age < 40 (N = 3,124, 42.1%) Ref 1.43 (0.64, 3.17) 2.01 (0.85, 4.78) 1.30 (0.64, 2.64) 0.080  Age 40–60 (N = 2,965, 43.9%) Ref 1.03 (0.64, 1.64) 0.79 (0.49, 1.27) 0.70 (0.37, 1.32)  Age ≥ 60 (N = 1,284, 14.0%) Ref 1.19 (0.69, 2.04) 0.92 (0.49, 1.73) 0.36 (0.14, 0.88)  **Sex**  Male (N = 3,778, 51.4%) Ref 1.60 (0.91, 2.83) 1.53 (0.95, 2.46) 0.98 (0.54, 1.76) 0.198  Female (N = 3,595, 48.6%) Ref 0.99 (0.66, 1.47) 0.73 (0.49, 1.06) 0.67 (0.44, 1.02)  **Race**  AA (N = 1,530, 10.5%) Ref 1.18 (0.61, 2.29) 1.12 (0.58, 2.16) 0.85 (0.50, 1.42) 0.859  Non-AA (N = 5,843, 89.5%) Ref 1.18 (0.79, 1.77) 1.04 (0.75, 1.43) 0.77 (0.53, 1.11)  **Diabetes**  Diabetes (N = 939, 9.6%) Ref 1.57 (0.73, 3.38) 0.86 (0.42, 1.78) 0.49 (0.20, 1.21) 0.321  No diabetes (N = 6,434, 90.4%) Ref 1.05 (0.72, 1.53) 1.12 (0.76, 1.64) 0.89 (0.62, 1.28)  **Hypertension**  Hypertension (N = 2,894, 35.5%) Ref 1.12 (0.67, 1.85) 0.87 (0.56, 1.38) 0.66 (0.37, 1.18) 0.534  No hypertension (N = 4,479, 64.5%) Ref 1.25 (0.71, 2.18) 1.25 (0.75, 2.06) 0.93 (0.58, 1.49) |

*Abbreviations:* *eGFR* estimated Glomerular Filtration Rate, *AA* African American.

**p*-value for interaction was calculated by treating urine osmolality quartiles and age groups as continuous variables based on the median values.

†Decreased eGFR was defined by eGFR < 60 mL/min/1.73m^2^. Albuminuria was defined by ACR ≥ 30 mg/gm.

°*Adjusted* for age, sex, race/ethnicity, education, family income-to-poverty ratio, smoking, BMI, hypertension, coronary heart disease, and dietary sodium intake in the subgroups of diabetes. Adjusted for age, sex, race/ethnicity, education, family income-to-poverty ratio, smoking, BMI, diabetes, coronary heart disease, and dietary sodium intake in the subgroups of hypertension.

**Supplement Figure SF1**

**A** **B**


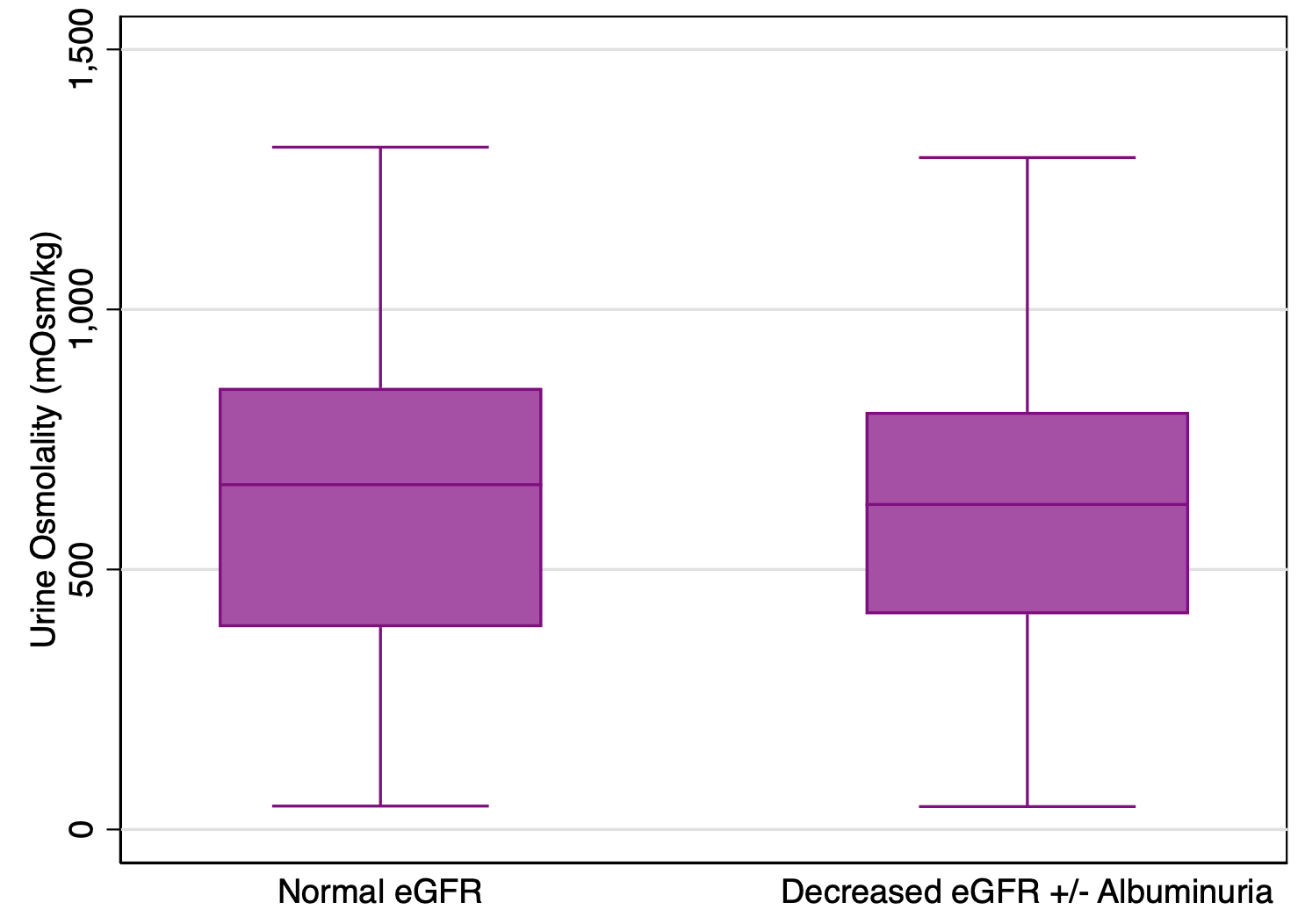

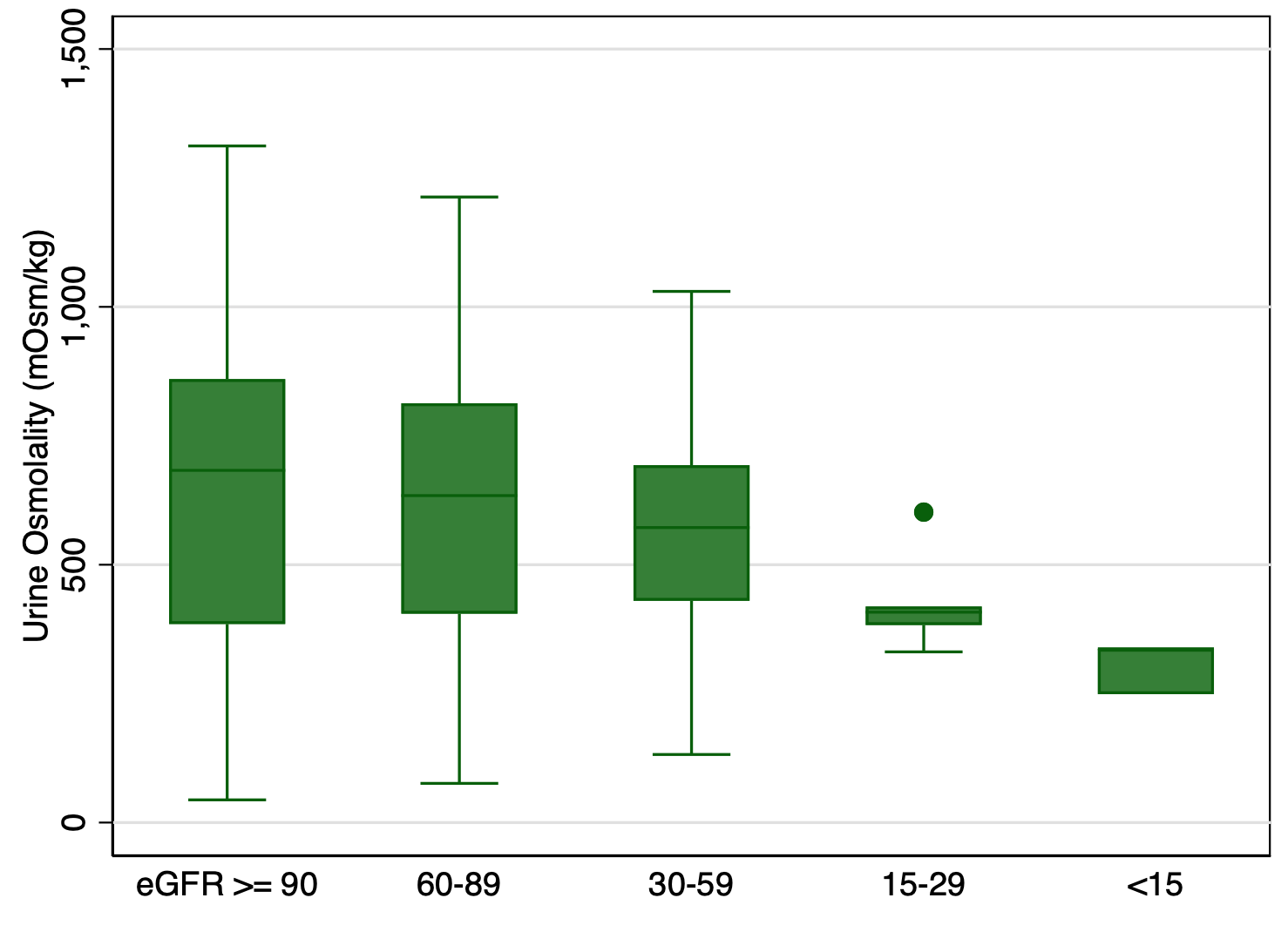

Supplement: Supplementary file 1 — Additional file 1: Table ST1. Subgroup analyses examining the association between urine osmolality quartiles with decreased eGFR and/or albuminuria. Figure SF1. A Distribution of urine osmolality by the presence of decreased eGFR and/or albuminuria, B Distribution of urine osmolality by eGFR levels. [file 12882_2021_2478_MOESM1_ESM.docx]
